# The Use of Propofol as a Sedative Agent in Gastrointestinal Endoscopy: A Meta-Analysis

**DOI:** 10.1371/journal.pone.0053311

**Published:** 2013-01-08

**Authors:** Daorong Wang, Chaowu Chen, Jie Chen, Yaxiang Xu, Lu Wang, Zhen Zhu, Denghao Deng, Juan Chen, Aihua Long, Dong Tang, Jun Liu

**Affiliations:** 1 Department of Gastrointestinal Surgery, Subei People’s Hospital of Jiangsu Province (Clinical Medical College of Yangzhou University), Yangzhou, Jiangsu Province, People’s Republic of China; 2 Department of Gastroenterology, Subei People’s Hospital of Jiangsu Province (Clinical Medical College of Yangzhou University), Yangzhou, Jiangsu Province, People’s Republic of China; Università Vita-Salute San Raffaele, Italy

## Abstract

**Objectives:**

To assess the efficacy and safety of propofol sedation for gastrointestinal endoscopy, we conducted a meta-analysis of randomized controlled trials (RCTs) comparing propofol with traditional sedative agents.

**Methods:**

RCTs comparing the effects of propofol and traditional sedative agents during gastrointestinal endoscopy were found on MEDLINE, the Cochrane Central Register of Controlled Trials, and EMBASE. Cardiopulmonary complications (i.e., hypoxia, hypotension, arrhythmia, and apnea) and sedation profiles were assessed.

**Results:**

Twenty-two original RCTs investigating a total of 1,798 patients, of whom 912 received propofol only and 886 received traditional sedative agents only, met the inclusion criteria. Propofol use was associated with shorter recovery (13 studies, 1,165 patients; WMD –19.75; 95% CI –27.65, 11.86) and discharge times (seven studies, 471 patients; WMD –29.48; 95% CI –44.13, –14.83), higher post-anesthesia recovery scores (four studies, 503 patients; WMD 2.03; 95% CI 1.59, 2.46), better sedation (nine studies, 592 patients; OR 4.78; 95% CI 2.56, 8.93), and greater patient cooperation (six studies, 709 patients; WMD 1.27; 95% CI 0.53, 2.02), as well as more local pain on injection (six studies, 547 patients; OR 10.19; 95% CI 3.93, 26.39). Effects of propofol on cardiopulmonary complications, procedure duration, amnesia, pain during endoscopy, and patient satisfaction were not found to be significantly different from those of traditional sedative agents.

**Conclusions:**

Propofol is safe and effective for gastrointestinal endoscopy procedures and is associated with shorter recovery and discharge periods, higher post-anesthesia recovery scores, better sedation, and greater patient cooperation than traditional sedation, without an increase in cardiopulmonary complications. Care should be taken when extrapolating our results to specific practice settings and high-risk patient subgroups.

## Introduction

The number of gastrointestinal endoscopic procedures performed in the United States has increased between two- and four-fold during the last few years, and more than 98% of routine endoscopies are now performed with sedation [1]. The goals of sedation are analgesia, amnesia, control of patient behavior during the procedure, ability to complete the endoscopy and prompt patient recovery to the pretreatment level of consciousness [2] However, the need for sedation during these procedures is not generally accepted. Traditional sedative agents, such as benzodiazepines, have shown variable outcomes because of unstable levels of sedation [3], which can lead to patient discontent and difficulties in performing the endoscopy.

Propofol, an ultra-short-acting sedative agent with a rapid recovery profile, has been used extensively for gastrointestinal endoscopy [4]. Several empirical statements and reports have compared propofol to traditional sedative agents for gastrointestinal endoscopy and suggested that the benefits of propofol supersede those of traditional sedative agents [4], [5]. A number of small randomized controlled trials (RCTs) have evaluated the efficacy of propofol for gastrointestinal endoscopy compared to traditional sedative agents, with varying results [3], [6]–[27]. However, because of inadequate sample sizes, these trials may have individually failed to detect significant differences in cardiopulmonary complications and other components of the sedation profile. A 2005 meta-analysis of 12 RCTs summarized the potential benefits of propofol sedation during gastrointestinal endoscopy by comparing the cardiopulmonary complications (i.e., hypoxia, hypotension, arrhythmia, and apnea) between propofol and traditional sedative agents, but without efficacy endpoints [28]. They concluded that propofol sedation only during colonoscopy appears to have lower odds of cardiopulmonary complications compared with traditional sedative agents. They concluded that propofol sedation only during colonoscopy appears to have lower odds of cardiopulmonary complications compared with traditional agents. The primary aim of this review was to update current findings on the safety and efficacy of propofol for gastrointestinal endoscopy, incorporating 10 more recent RCTs and including efficacy endpoints.

## Methods

### Search Strategy

The PubMed, Ovid, MEDLINE and EMBASE databases were searched, in addition to the Cochrane Central Register of Controlled Trials, to locate articles (1966 to June 2012) on propofol sedation (PS) for gastrointestinal endoscopy in adults. The search terms *colonoscopy*, *Diprivan*, *double-balloon endoscopy* (DBE), *endoscopic retrograde cholangiopancreatography* (ERCP), *endoscopic ultrasonography* (EUS), *esophagogastroduodenoscopy* (EGD), *propofol*, *sedation*, *sigmoidoscopy* and *upper gastrointestinal endoscopy* were used. References, lists of retrieved articles, reviews and meta-analyses were then scanned for additional articles. Internet search engines were also used to perform a manual search for abstracts from international meetings, which were then downloaded and studied.

### Study Selection

RCTs met the inclusion criteria if they involved propofol as the sole sedative agent for gastrointestinal endoscopy in adult patients (i.e., those 18 years and older) and used other sedative agents (without propofol) for control. All studies that did not use propofol as the sedative agent, or used propofol with other sedative agents simultaneously, were excluded. Studies that could not provide actual frequencies of complications (i.e., those that gave only percentages of complications or percentage decline in complications) were also excluded. Both full-length publications and abstract publications were selected. Letters, reviews without original data, non-English papers and animal studies were excluded. If any doubt of suitability remained after the abstract was examined, the full manuscript was obtained.

### Data Extraction

Two review authors assessed the methodological quality of potentially eligible trials, without consideration of the results. All the data extracted independently by the two authors, and then cross-checked between them to rule out the discrepancy. Pooled data were examined for cardiopulmonary complications (hypoxia, hypotension, arrhythmia, and apnea) and other aspects of the sedation profile, including recovery time, discharge time, postanesthesia recovery score (PARS), sedation level, and patient cooperation. Complications were defined objectively as follows: hypoxia as a drop in oxygen saturation below 90%, hypotension as a drop in systolic blood pressure below 90 mmHg, arrhythmia as a heart rhythm different from the patient’s usual rhythm, and apnea as cessation of respiratory activity for more than 10 seconds. These complications were not mutually exclusive, and more than one could potentially occur in the same patient. All RCTs were assigned a quality score based on the Jadad scale [29], with 5 indicating the highest quality and 0 indicating the poorest quality. Disagreements were discussed by the authors and resolved by consensus.

### Statistical Analysis

The software package RevMan 4.2 provided by the Cochrane Collaboration was used for analysis. Separate analyses were performed for each outcome using an odds ratio (OR) or weighted mean difference (WMD), at the same time the subgroup analyses was also performed according to the results of different groups (group of ERCP, EGD, and Colonoscopy). Depending on the absence or presence of significant heterogeneity, meta-analysis was conducted using the random-effects model or the fixed-effect model. Heterogeneity was assessed using the χ^2^ test with significance set at *P*<0.10. The *I* ^2^ statistic was used to quantify heterogeneity. Using accepted guidelines [30], an *I* ^2^ of 0% to 40% was considered to exclude heterogeneity, an *I* ^2^ of 30% to 60% to represent moderate heterogeneity, an *I* ^2^ of 50% to 90% to represent substantial heterogeneity, and an *I* ^2^ of 75% to 100% to represent considerable heterogeneity; statistical significance was represented by a 95% confidence interval (CI) and a *P*-value of <0.05. Publication bias was tested by funnel plots and Egger’s linear regression using Stata 11.0 software (Stata Corporation, College Station, TX, USA), and *P*<0.05 was considered significant.

## Results

### Search Results

**Figure 1:**
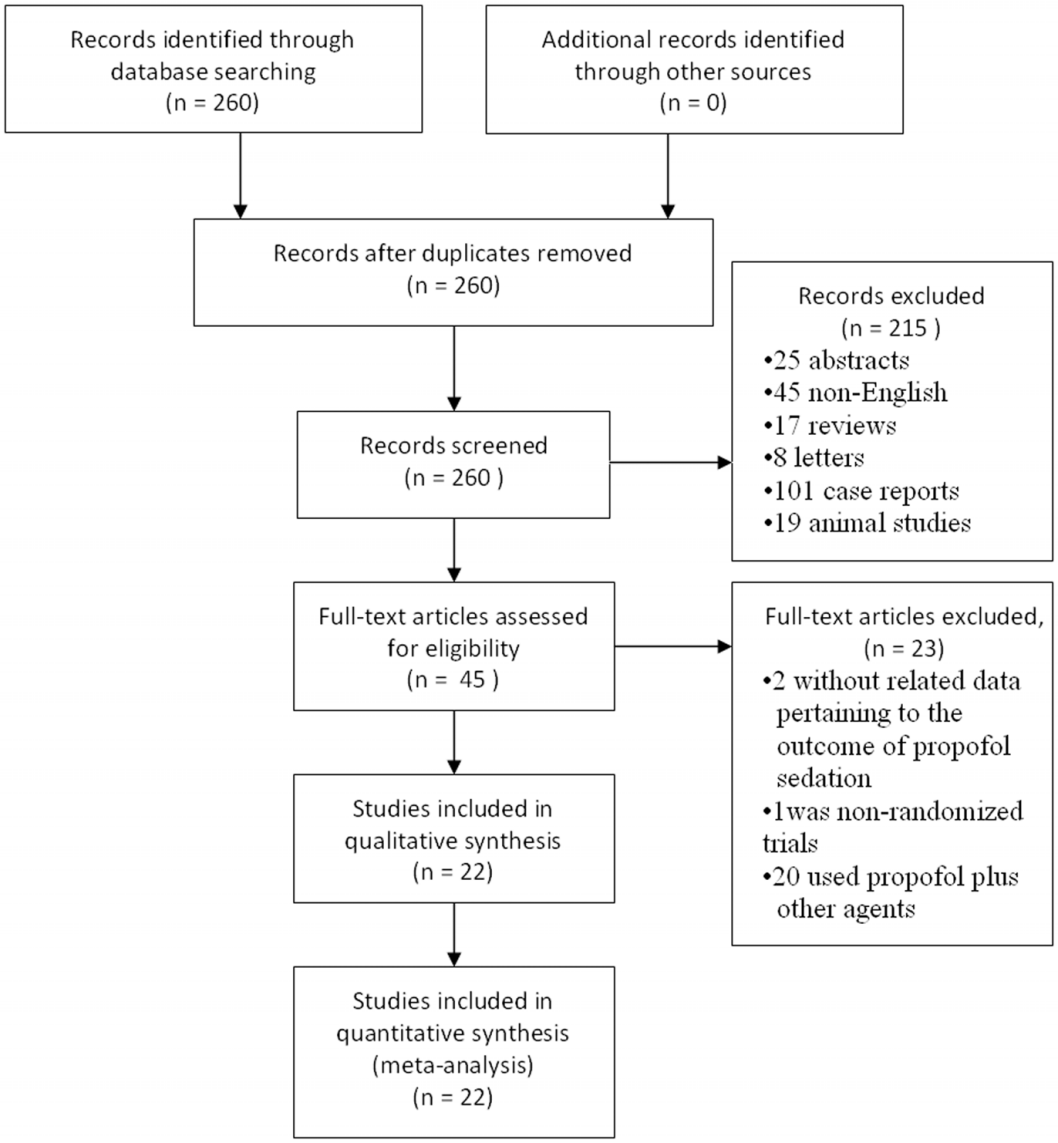
Flow chart of study selection.

A total of 260 references were identified from medical journal databases. Upon examination of the abstracts, 215 articles were rejected based on the rejection criteria outlined in Figure 1. Study of the complete manuscripts for the 45 remaining articles led to elimination of two papers that contained no data pertaining to the outcome of PS for gastrointestinal endoscopy, one paper that described nonrandomized trials and 20 papers that involved the use of propofol plus other sedative agents simultaneously in the same group. The remaining 22 nonduplicated RCTs that compared PS with traditional sedation (TS) were included in the meta-analysis.

### Characteristics of the Selected RCTs

Characteristics of the 22 RCTs included in the meta-analysis are summarized in Table S1. These studies were published between 1991 and 2011 and investigated a total of 1,798 patients, 912 of whom received PS and 886 of whom received TS. Eight of the studies involved EGD (425 patients), four involved colonoscopy (308 patients), six involved ERCP (663 patients), one involved EUS (80 patients), and the other three involved a mixture of gastrointestinal endoscopy procedures (322 patients). The large sample sizes allowed analysis of EGD, colonoscopy, and ERCP procedure subgroups. The traditional sedative agents, midazolam, midazolam plus meperidine, meperidine plus scopolamine, midazolam plus fentanyl and midazolam plus pentazocine, were used for controls. Sixteen of the selected RCTs had Jadad scores greater than or equal to 3, which suggested a good study design and high report quality.

### Meta-analysis Results

#### Cardiopulmonary complications

**Figure 2:**
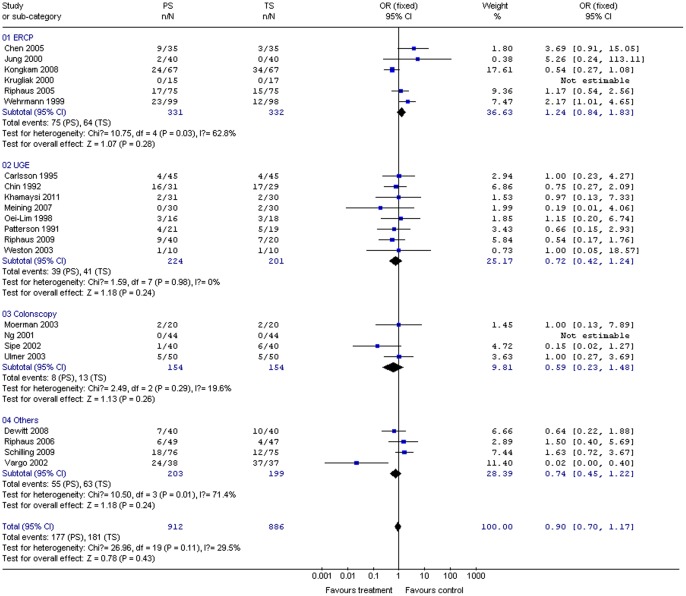
Forest plot demonstrating the overall number of complications of PS vs. TS for gastrointestinal endoscopy.

Cardiopulmonary complications were recorded in a consistent manner across most of the studies; however, the apneas recorded in four studies could not be analyzed further by procedure subgroups. Meta-analysis results indicated that PS during gastrointestinal endoscopy carried similar odds of cardiopulmonary complications compared to TS (*P*>0.05 for test effect; Table 1). Pooled OR of overall cardiopulmonary complications also showed no difference between PS and TS groups for any procedure subgroup or for all procedures combined (Fig. 2). Moreover, there are 5 studies with high-risk patients included in our meta-analysis. 141 cirrhotic patients were reported in 3 studies [12], [17], [22], in which 81 patients received PS and 60 patients received TS. 301 octogenarians patients with high comorbidity were also reported in 2 studies [23], [26], in which 151 patients received PS and 150 patients received TS. After analysis of the high-risk patients, our meta-analysis results indicated that PS during gastrointestinal endoscopy had similar odds of cardiopulmonary complications compared to TS in high-risk patients (P>0.05 for test effect). Pooled OR of overall cardiopulmonary complications also showed no difference between PS and TS groups for high-risk patients (Table 2).

**Table 1:** OR and 95% CI of cardiopulmonary complications for PS vs. TS during gastrointestinal endoscopy in all of the patients.

Complications	Procedures	Studies included	No. of patients	Odds ratio	95% CI	P-value for heterogeneity
Hypoxia	Combined	21	1719	0.79	0.58–1.09	0.97
	Subgroups					
	ERCP	5	584	0.91	0.55–1.49	0.57
	EGD	8	435	0.71	0.39–1.30	1.00
	Colonoscopy	4	308	1.00	0.14–7.22	0.34
Hypotension	Combined	20	1653	1.46	0.92–2.31	0.66
	Subgroups					
	ERCP	5	593	1.69	0.82–3.50	0.62
	EGD	8	425	0.85	0.21–3.35	0.60
	Colonoscopy	4	308	0.58	0.17–1.94	0.22
Arrhythmia	Combined	19	1572	0.93	0.52–1.65	0.46
	Subgroups					
	ERCP	5	593	0.84	0.38–1.88	0.23
	EGD	7	365	0.87	0.25–3.03	0.29
	Colonoscopy	4	308	0.66	0.11–4.02	0.22
Apnea	Combined	11	947	0.60	0.27–1.32	0.66

**Table 2:** OR and 95% CI of cardiopulmonary complications for PS vs. TS during gastrointestinal endoscopy in high-risk patients.

Complications	Studies included	No. of patients	Odds ratio	95% CI	P-value for heterogeneity
Hypoxia	5	442	1.06	0.56–2.02	0.93
Hypotension	5	442	1.51	0.63–3.60	0.82
Arrhythmia	5	442	0.90	0.37–2.17	0.50
Overall	5	442	1.14	0.70–1.85	0.68

#### Procedure duration

**Figure 3:**
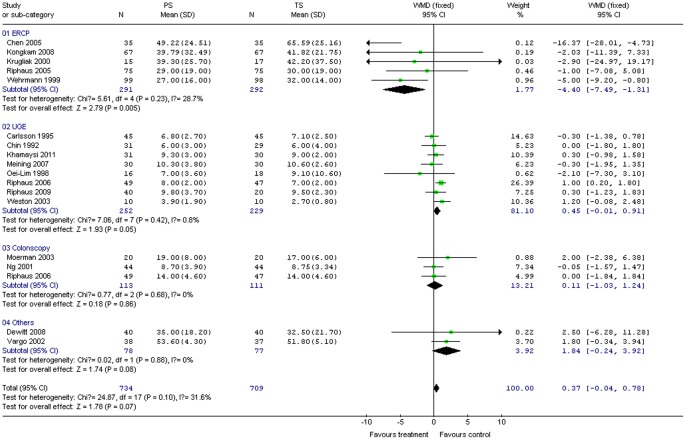
Forest plot demonstrating procedure duration with PS vs. TS for gastrointestinal endoscopy.

Comparison of procedure duration between propofol and control groups was measured for all procedures in 18 studies (1,443 patients). No significant difference was discovered on pooling the results for these two groups (WMD 0.37; 95% CI –0.04, 0.78; *P = *0.07). The χ^2^ and *I* ^2^ were 24.87 (*P = *0.10) and 31.6%, respectively, suggesting the absence of heterogeneity among the studies (Fig. 3). Subgroup analysis indicated that PS for ERCP was associated with significantly shorter procedure duration compared with TS (WMD –4.40; 95% CI –7.49, –1.31; *P = *0.005); the χ^2^ and *I* ^2^ were 5.61 (*P* = 0.23) and 28.7%, respectively, indicating the absence of heterogeneity among the studies (Fig. 3).

#### Recovery time

**Figure 4:**
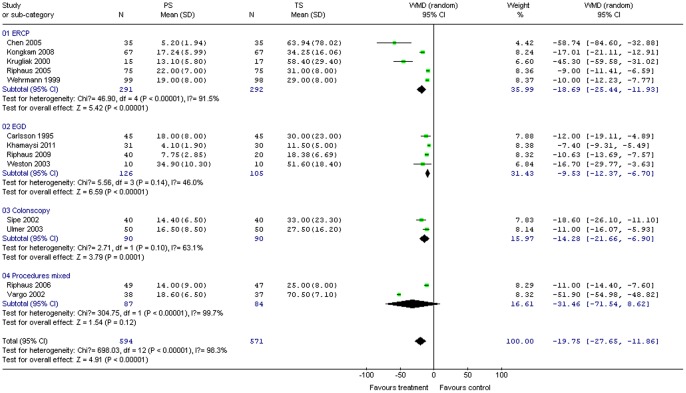
Forest plot demonstrating recovery time with PS vs. TS for gastrointestinal endoscopy.

Thirteen studies (1,165 patients) provided data on recovery time. PS significantly reduced mean recovery time compared with TS for all procedures combined (WMD –19.75; 95% CI –27.65, –11.86; *P*<0.01). The χ^2^ and *I* ^2^ were 698.03 (*P*<0.10) and 98.3%, respectively, indicating heterogeneity among the studies (Fig. 4). Subgroup analysis indicated significantly shorter recovery time with PS compared to TS for all groups (Fig. 4).

#### Discharge time

**Figure 5:**
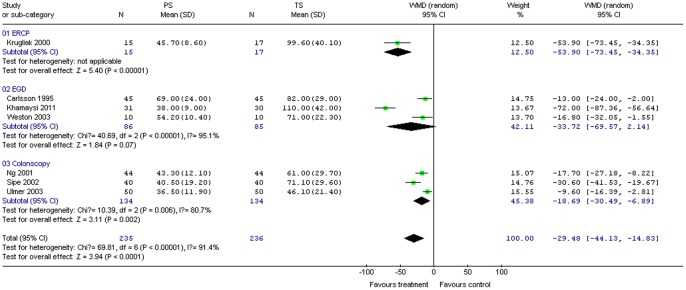
Forest plot demonstrating discharge time with PS vs. TS for gastrointestinal endoscopy.

Seven studies (249 patients) provided data on discharge time. PS significantly reduced mean discharge time compared with TS for all procedures combined (WMD –29.48; 95% CI –44.13, –14.83; *P*<0.01). The χ^2^ and *I* ^2^ were 69.81 (*P*<0.10) and 91.4%, respectively, indicating heterogeneity among the studies (Fig. 5). Subgroup analysis indicated significantly shorter recovery time for PS compared to TS for colonoscopy (three studies, 268 patients; WMD –18.69; 95% CI –30.49, –6.89; *P = *0.002); χ^2^ and *I* ^2^ were 10.39 (*P = *0.006) and 80.7%, respectively, indicating heterogeneity among the studies. For the ERCP subgroup, only one study (32 patients) reported discharge time, revealing a trend toward shorter discharge time following PS compared to TS. While no significant difference in discharge time arose between propofol and TS groups for EGD (three studies, 171 patients; WMD –33.72; 95% CI –69.57, 2.14; *P = *0.07), the χ^2^ and *I* ^2^ were 40.69 (*P*<0.01) and 95.1%, respectively, indicating heterogeneity among the studies (Fig. 5).

#### Post-anesthesia recovery score (PARS)

**Figure 6:**
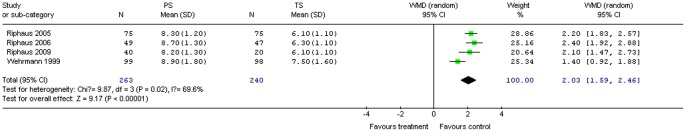
Forest plot demonstrating PARS with PS vs. TS for gastrointestinal endoscopy.

Four studies (503 patients) provided data on recovery time; all of them found a higher PARS in the PS group than in the TS group (WMD 2.03; 95% CI 1.59, 2.46; *P*<0.01). The χ^2^ and *I* ^2^ were 9.87 (*P = *0.02) and 69.6%, respectively, suggesting heterogeneity among the studies (Fig. 6).

#### Sedation level

**Figure 7:**
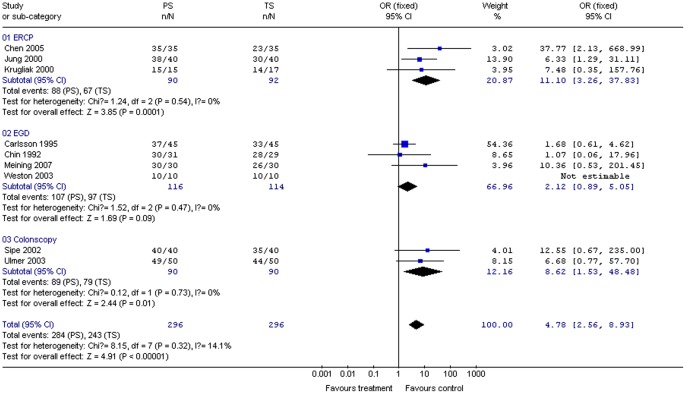
Forest plot demonstrating sedation level with PS vs. TS for gastrointestinal endoscopy.

Correct sedation was defined as the absence of patient resistance to the procedure. Nine studies (592 patients) provided data on sedation level. Propofol administration significantly increased the sedation level compared to use of traditional sedative agents for all procedures combined (OR 4.78; 95% CI 2.56, 8.93; *P*<0.01); χ^2^ and *I* ^2^ were 8.15 (*P = *0.32) and 14.1%, respectively, indicating the absence of heterogeneity among the studies (Fig. 7). Subgroup analysis indicated that propofol provided significantly better sedation than traditional sedative agents for ERCP (OR 11.10; 95% CI 3.26, 37.83; *P = *0.0001) and colonoscopy (OR 8.62; 95% CI 1.53, 48.48; *P = *0.01), but no difference was found for upper gastrointestinal endoscopy (OR 2.12; 95% CI 0.89, 5.05; *P = *0.09). The χ^2^ and *I* ^2^ were respectively 1.24 (*P* = 0.54) and 0% for ERCP, 0.12 (*P* = 0.73) and 0% for colonoscopy, and 1.52 (*P* = 0.47) and 0% for upper gastrointestinal endoscopy, indicating the absence of heterogeneity among the studies (Fig. 7).

#### Patient cooperation

**Figure 8:**
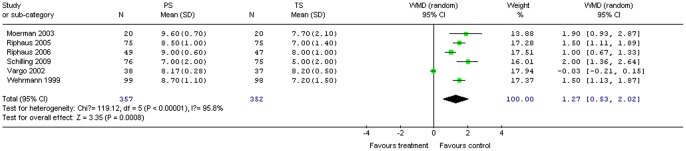
Forest plot demonstrating patient cooperation with PS vs. TS for gastrointestinal endoscopy.

Patient cooperation was assessed by patient response to the visual analogue scale (VAS) [3], [8], [21], [23], [26], [27]. Six studies (709 patients) provided data on patient cooperation. PS significantly increased patient cooperation compared with TS for all procedures combined (WMD 1.27; 95% CI 0.53, 2.02; *P = *0.0008). The χ^2^ and *I* ^2^ were 119.12 (*P*<0.10) and 95.8%, respectively, which indicated heterogeneity among the studies (Fig. 8).

#### Local pain on injection

**Figure 9:**
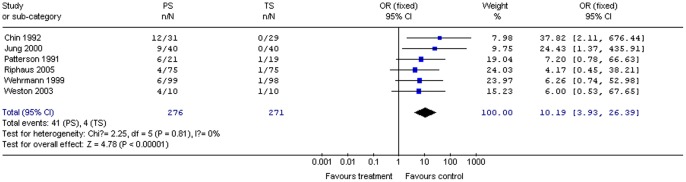
Forest plot demonstrating local pain on injection with PS vs. TS for gastrointestinal endoscopy.

Six studies (547 patients) reported data on pain associated with sedative administration. PS significantly increased local pain on injection compared with TS for all procedures combined (OR 10.19; 95% CI 3.93, 26.39; *P*<0.01). The χ^2^ and *I* ^2^ were 2.25 (*P = *0.81) and 0%, respectively, indicating the absence of heterogeneity among the studies (Fig. 9).

#### Amnesia

**Figure 10:**
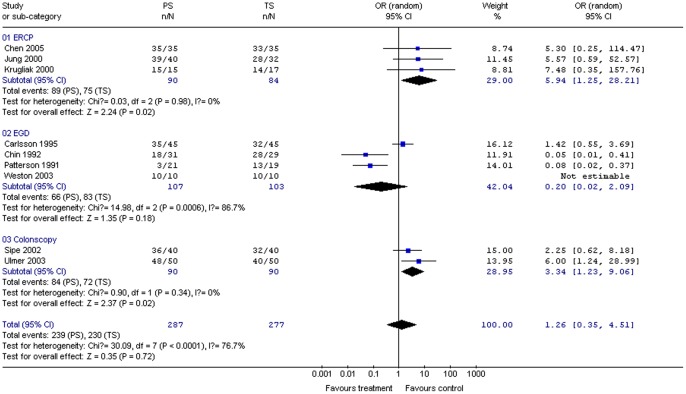
Forest plot demonstrating amnesia with PS vs. TS for gastrointestinal endoscopy.

Nine studies (564 patients) compared amnesia between propofol and control groups. Although all of them showed a trend toward more amnesia in the propofol group, the pooled mean difference between propofol and control groups was 1.26 (95% CI 0.35, 4.51), suggesting a statistically non-significant difference between the two groups. The χ^2^ and *I* ^2^ were 30.09 (*P*<0.10) and 76.7%, respectively, indicating heterogeneity among the studies (Fig. 10). Subgroup analysis indicated that PS produced significantly more amnesia than TS for ERCP (OR 5.98; 95% CI 1.26, 28.40; *P = *0.02) and colonoscopy (OR 3.50; 95% CI 1.32, 9.31; *P = *0.01). The χ^2^ and *I* ^2^ were respectively 0.03 (*P* = 0.98) and 0% for ERCP, and 0.90 (*P* = 0.34) and 0% for colonoscopy, indicating the absence of heterogeneity among the studies (Fig. 10).

#### Pain during the procedure

**Figure 11:**
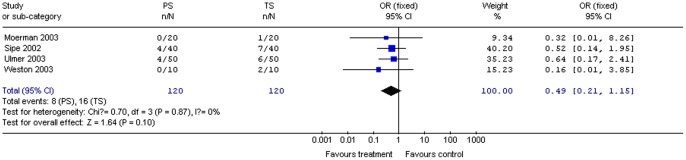
Forest plot demonstrating procedural pain with PS vs. TS for gastrointestinal endoscopy.

Four studies (240 patients) provided data on pain during the procedure. Pooling the results showed no significant difference in procedural pain between propofol and control groups for all procedures combined (OR 0.49; 95% CI 0.21, 1.15; *P = *0.10). The χ^2^ and *I* ^2^ were 0.70 (*P = *0.87) and 0%, respectively, indicating the absence of heterogeneity in the studies (Fig. 11).

#### Patient satisfaction

**Figure 12:**
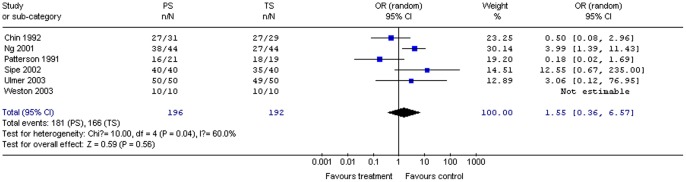
Forest plot demonstrating patient satisfaction with PS vs. TS for gastrointestinal endoscopy.

Six studies (388 patients) provided data on patient satisfaction. Pooling the results for propofol and control groups revealed no significant difference in patient satisfaction (OR 1.55; 95% CI 0.36, 6.57; *P = *0.56). The χ^2^ and *I* ^2^ were 10.0 (*P = *0.04) and 60%, respectively, suggesting moderate heterogeneity among the studies (Fig. 12).

### Publication Bias

**Figure 13:**
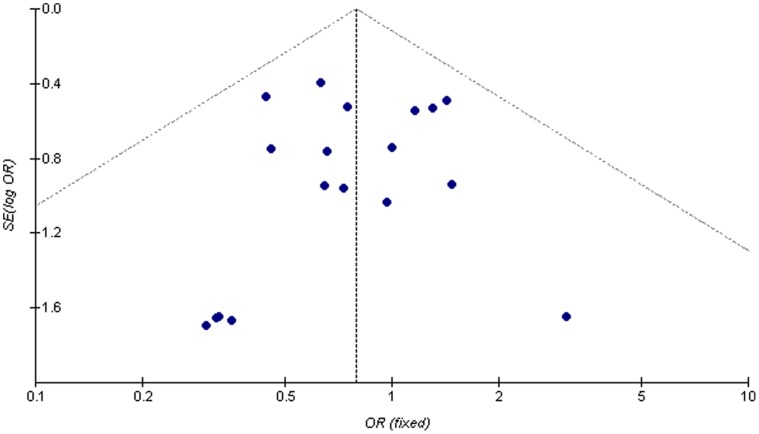
Funnel plot assessing publication bias. No publication bias was noted. SE, standard error.

Funnel plotting and Egger’s testing were performed to assess the publication bias of the studies used. Funnel plot analysis was performed using the occurrence of hypoxia as an index, and the funnel plot of the 21 studies appeared to be symmetrical (Fig. 13). In overall studies, no significant publication bias (*P*<0.05) was found (data not shown).

## Discussion

A growing amount of evidence demonstrates that propofol has been used safely and effectively in gastrointestinal endoscopy [6], [7], [24], [25], [26], [27], even for high-risk patients [4], [12], [17], [22], [23], [26]. A previous meta-analysis published on this topic compared only the risks of propofol with those of traditional sedative agents. While that study concluded that PS appeared to yield lower odds of cardiopulmonary complications during colonoscopy compared to traditional sedative agents but similar risks for other procedures [28], subgroup analysis in our investigation indicated that PS did not reduce the odds of complications during colonoscopy compared to traditional sedative agents. Our meta-analysis showed that the incidence of cardiopulmonary complications during gastrointestinal endoscopy with propofol was not significantly different from the incidence of complications with TS, which suggested that PS is as safe as TS for all gastrointestinal endoscopy procedures.

In addition to assessing cardiopulmonary complications, our study analyzed many aspects of the sedation profile and concluded that PS results in faster recovery and discharge times, higher PARS, better sedation level, and greater patient cooperation compared to TS. In gastrointestinal endoscopy units, where throughput is limited by the availability of recovery room resources, propofol may therefore provide a distinct advantage by facilitating faster turnover of patients to meet increasing demands for gastrointestinal endoscopy [31].

The studies included in this review assessed and reported amnesia. Although there was no significant difference in amnesia between PS and TS for all gastrointestinal endoscopy procedures combined, significantly greater amnesia was found for ERCP and colonoscopy with propofol use. Given the amnestic but not analgesic properties of propofol, the pain control provided by propofol alone may be a reflection of patients’ inability to remember pain. Alternatively, propofol may produce deeper levels of sedation and anesthesia, thereby suppressing physiological brain functions, including the ability to register pain [32]. If the observed pain control is due to amnesia, autonomic responses to pain may still manifest during the procedure itself [31].

Although most of the studies included in our meta-analysis required healthy participants, high-risk patients were also studied, including patients age 80 and older with high comorbidity [23], [26], patients with cirrhosis [17], [22], and patients with subclinical hepatic encephalopathy [12]. A study involving sick patients found a complication rate higher than the rates determined in earlier investigations of healthy patients [33]. Although our meta-analysis indicated no difference in cardiopulmonary complications between high-risk patients who were given propofol and those who were given traditional sedatives, the safety of PS in high-risk patients needs confirmation by large-sample RCTs.

Caution should be used when extrapolating our findings to different, specific practice situations. First, the administrators included in our study are different as follows: nurses, gastroenterologists, endoscopists and physicians, all of whom might have different training level. And the methods of administration are different as follows: bolus injection, per-kilogram infusion, patient-controlled sedation (PCS), and target controlled infusion (TCI), all of which might also have different level of sedation [34], [35], [36], [37]. Second, the patients in our study included some high-risk patients such as octogenarians patients with high comorbidity and liver cirrhotic patients, these patients might suffer more adverse events compare to the healthy participants. Last, different drugs may cause different adverse events. Propofol is a short-acting anesthetic agent that has a favorable pharmacokinetic profile in comparison to the benzodiazepines and opioids with regard to rapid induction of sedation, faster recovery, and equivalent levels of amnesia [12]. Midazolam is a benzodiazepine depressant of the central nervous system that is commonly used in synergy with the opioid analgesic meperidine for conscious sedation during GI endoscopy [38]. This combination is widely used because of its short-acting sedative, anxiolytic, and amnesic properties [39], but with the following shortage: a delay of several minutes after injection before the drugs exert their effect, lingering sedative effects that delay discharge, significant cost because of monitoring and prolonged recovery, and morbidity and mortality as a result of respiratory depression [9]. Therefore, the optimization of propofol administration methods for gastrointestinal procedures need further study.

Furthermore, as propofol has limited analgesic effect and higher doses are often required, when it is used as a single agent for gastrointestinal endoscopy, resulting in higher sedation levels. Previous studies have demonstrated that, compared with propofol alone, propofol combined with traditional sedative agents that targeted to moderate sedation is associated with a lower risk of complications, better patient cooperation, superior patient satisfaction and shorter recovery times in gastrointestinal endoscopy [40], [41]. Thus use of propofol in combination with other agents might be preferable to propofol alone. The combination may be easier to manage due to lower sedation levels and ability to reverse some of the sedation with the use of reversal agents for narcotics and/or benzodiazepines [31]. While, other studies suggested that propofol in combination with other agents offered no benefits compared with the use of propofol alone [42], [43], [44], [45]. So the safe of propofol in combination with other agents is also need large number RCTs to assess.

Adequate training in administration of sedative agents, monitoring during sedation, and the ability to recover patients from deeper levels of sedation have been receiving more attention. All individuals involved in the administration of propofol, including nurses, gastroenterologists, endoscopists and physicians, are trained and certified in advanced cardiac life support. Recently, electroencephalogram (EEG) and bispectral index (BIS) monitoring have been reported to yield precise measurement of sedation level, facilitating more effective titration of propofol dosage and faster patient recovery for ERCP [5], [46]. Methods for promoting the safety and efficiency of PS still need further study.

Our meta-analysis pooled all available data from published RCTs investigating propofol sedation during gastrointestinal endoscopy, substantially reducing type II error. This meta-analysis has presented of a lot of outcomes; those with heterogeneity should be viewed with some caution. However, the heterogeneity among the studies for many of the outcomes (recovery time, discharge time, patient cooperation, PARS, amnesia, and patient satisfaction) can largely be explained by subgroup and sensitivity analyses. The heterogeneity among the studies should be taken into account in interpreting the results. Firstly, different varieties of traditional sedative agents were used for control. Secondly, sedation was administered variously by anesthetists, ICU physicians, endoscopists, gastroenterologists, nurses and unspecified physicians. In one study, sedation was even controlled by the patients themselves [13]. Thirdly, data were not complete for all outcomes. Last, three studies reported combined data for all gastrointestinal endoscopy procedures, rendering subgroup analysis impossible. However there was no heterogeneity for procedure duration, sedation level, local pain on injection, pain during endoscopy or any of the cardiopulmonary complications, with propofol yielding better sedation and no difference in cardiopulmonary complications compared to traditional sedative agents.

In conclusion, this meta-analysis demonstrates that propofol is a safe and effective sedative agent for all gastrointestinal endoscopy procedures, facilitating a faster recovery time, higher PARS, better sedation level, and greater patient cooperation compared to traditional sedative agents without increasing the risk of cardiopulmonary complications.

## Supporting Information

Figure S1 **PRISMA 2009 Flow Diagram.**
(DOC)

Table S1 **Summary of randomized controlled trials included in the meta-analysis.**
(DOC)

Table S2 **PRISMA 2009 Checklist.**
(DOC)
