# Supplementary material for: The Use of Propofol as a Sedative Agent in Gastrointestinal Endoscopy: A Meta-Analysis
Source: PLoS One. 2013 Jan 8;8(1):e53311. doi: 10.1371/journal.pone.0053311 (PMC3540096; doi:10.1371/journal.pone.0053311)
Supplement: Figure S1 — PRISMA 2009 Flow Diagram. (DOC) [file pone.0053311.s001.doc]

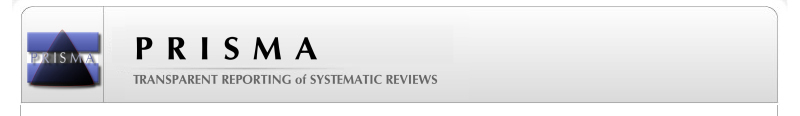
**PRISMA 2009 Flow Diagram**

**Screening**

**Included**

**Eligibility**

**Identification**

Records identified through database searching
(n = 260)

Additional records identified through other sources
(n = 0)

Records after duplicates removed
(n = 260)

Records screened
(n = 260 )

Records excluded
(n = 215 )

•25 abstracts

•45 non-English

•17 reviews

•8 letters

•101 case reports

•19 animal studies

Full-text articles assessed for eligibility
(n = 45 )

Full-text articles excluded, (n = 23)

•2 without related data pertaining to the outcome of propofol sedation

•1was non-randomized trials

•20 used propofol plus other agents

Studies included in qualitative synthesis
(n = 22)

Studies included in quantitative synthesis (meta-analysis)
(n = 22)
