# Supplementary material for: The Use of Propofol as a Sedative Agent in Gastrointestinal Endoscopy: A Meta-Analysis
Source: PLoS One. 2013 Jan 8;8(1):e53311. doi: 10.1371/journal.pone.0053311 (PMC3540096; doi:10.1371/journal.pone.0053311)
Supplement: Table S1 — Summary of randomized controlled trials included in the meta-analysis. (DOC) [file pone.0053311.s002.doc]

**Table S1:** Summary of randomized controlled trials included in the meta-analysis.

| **First author, year , Country** | **Administrator** | **Sedation** | **Procedure** | **N** | **HPX** | **HPT** | **AR** | **AP** | **RT** | PARS | **AM** | **SL** | **PC** | **Jadad score** |
| --- | --- | --- | --- | --- | --- | --- | --- | --- | --- | --- | --- | --- | --- | --- |
| Khamaysi^12^, 2011, Israel | Anaesthetist | Propofol | EGD | 31 | 2 | 0 | 0 |  | 4.1 ± 1.9 |  |  |  |  | 5 |
|  |  | Midazolam |  | 30 | 2 | 0 | 0 |  | 11.5 ± 5.0 |  |  |  |  |  |
| Riphaus^22^, 2009, Germany | Physician | Propofol | EGD | 40 | 3 | 3 | 3 |  | 7.75±2.85 | 8.2±1.3 |  |  |  | 3 |
|  |  | Midazolam |  | 20 | 2 | 2 | 3 |  | 18.38±6.69 | 6.1±1.1 |  |  |  |  |
| Schilling^23^, 2009, Germany | Nurse & physician | Propofol | ECRP/EUS/DBE | 58/15/3 | 9 | 4 | 5 | 0 |  |  |  |  | 7 ± 2 | 3 |
|  |  | Midazolam + meperidine |  | 53/19/3 | 7 | 2 | 3 | 0 |  |  |  |  | 5 ± 2 |  |
| Dewitt^18^, 2008, USA | Nurse & physician | Propofol | EUS | 40 | 3 | 4 | 0 |  |  |  |  |  |  | 3 |
|  |  | Midazolam +meperidine |  | 40 | 6 | 4 | 0 |  |  |  |  |  |  |  |
| Kongkam ^6^, 2008, Thailand | Gastroenterologist | Propofol | ERCP | 67 | 15 | 6 | 2 | 1 | 17.24±5.99 |  |  |  |  | 5 |
|  |  | Midazolam + meperidine |  | 67 | 21 | 6 | 7 | 0 | 34.25±16.06 |  |  |  |  |  |
| Meining^20^, 2007, Germany | Unknown | Propofol | EGD | 30 | 0 | 0 |  |  |  |  |  | 30 |  | 3 |
|  |  | Midazolam |  | 30 | 1 | 1 |  |  |  |  |  | 26 |  |  |
| Riphaus^21^, 2006, Germany | Physician | Propofol | Gastroscopy/  Colonoscopy | 49 | 3 | 3 |  |  | 14±9 | 8.7±1.3 |  |  | 9±0.6 | 4 |
|  |  | Midazolam+ pethidine |  | 47 | 2 | 2 |  |  | 25±8 | 6.3±1.1 |  |  | 8±1 |  |
| Chen^24^, 2005, China | ICU physician | Propofol | ERCP | 35 | 2 |  |  |  | 5.20 ± 1.94 |  | 35 | 35 |  | 2 |
|  |  | Meperidine+scopolamine |  | 35 | 3 |  |  |  | 63.94±78.02 |  | 33 | 23 |  |  |
| Riphaus ^26^, 2005, Germany | ICU physician | Propofol | ERCP | 75 | 8 | 6 | 3 |  | 22 ± 7 | 8.3±1.2 |  |  | 8.5±1.0 | 5 |
|  |  | Midazolam + meperidine |  | 75 | 7 | 4 | 4 |  | 31 ± 8 | 6.1±1.1 |  |  | 7.0±1.4 |  |
| Moerman^8^,2003, Belgium | Not known | Propofol | Colonoscopy | 20 | 0 | 0 | 0 | 2 |  |  |  |  | 9.6± 0.7 | 2 |
|  |  | Midazolam |  | 20 | 0 | 0 | 0 | 2 |  |  |  |  | 7.7± 2.1 |  |
| Ulmer^16^, 2003, USA | Endoscopists & Nurses | Propofol | Colonoscopy | 50 | 0 | 4 | 1 |  | 16.5±8.5 |  | 48 | 49 |  | 5 |
|  |  | Midazolam + fentanyl |  | 50 | 1 | 4 | 0 |  | 27.5±16.2 |  | 40 | 44 |  |  |
| Weston^17^, 2003, USA | Nurses | Propofol | EGD | 10 | 0 | 1 | 0 | 0 | 34.9±10.3 |  | 10 | 10 |  | 5 |
|  |  | Midazolam + meperidine |  | 10 | 1 | 0 | 0 | 0 | 51.6±18.4 |  | 10 | 10 |  |  |
| Sipe^9^,2002, USA | Nurses & physicians | Propofol | Colonoscopy | 40 | 1 | 0 | 0 | 0 | 14.4±6.5 |  | 36 | 40 |  | 4 |
|  |  | Midazolam + meperidine |  | 40 | 0 | 3 | 2 | 1 | 33.0±23.3 |  | 32 | 35 |  |  |
| Vargo^3^, 2002, USA | Gastroenterologist | Propofol | ECRP/EUS | 38 | 14 |  | 0 | 10 | 18.6 ± 6.5 |  |  |  | 8.17±0.28 | 5 |
|  |  | Midazolam + meperidine |  | 37 | 21 |  | 0 | 16 | 70.5 ± 7.1 |  |  |  | 8.2 ±0.5 |  |
| Ng^13^,2001, Singapore | Patient controlled | Propofol | Colonoscopy | 44 | 0 | 0 | 0 | 0 |  |  |  |  |  | 3 |
|  | Anaesthetist | Midazolam |  | 44 | 0 | 0 | 0 | 0 |  |  |  |  |  |  |
| Jung ^7^, 2000, Germany | Anaesthetist | Propofol | ERCP | 40 |  | 1 | 1 |  |  |  | 39/40 | 38 |  | 2 |
|  |  | Midazolam |  | 40 |  | 0 | 0 |  |  |  | 28/32 | 30 |  |  |
| Krugliak ^25^, 2000, Israel | Anaesthetist | Propofol | ERCP | 15 | 0 | 0 | 0 | 0 | 13.1 ± 5.8 |  | 15 | 15 |  | 5 |
|  |  | Midazolam |  | 17 | 0 | 0 | 0 | 0 | 58.4 ± 29.4 |  | 14 | 14 |  |  |
| Wehrmann ^27^, 1999, Germany | Physician | Propofol | ERCP | 99 | 11 | 7 | 5 | 0 | 19 ± 8 | 8.9±1.8 |  |  | 8.7 ± 1.1 | 4 |
|  |  | Midazolam+ pentazocine |  | 98 | 8 | 2 | 2 | 0 | 29 ± 8 | 7.5±1.6 |  |  | 7.2 ± 1.5 |  |
| Oei-Lim^14^, 1998, Netherlands | Anaesthetist | Propofol | EGD | 16 | 0 | 0 | 3 |  |  |  |  |  |  | 2 |
|  | Endoscopist | Midazolam |  | 18 | 1 | 0 | 2 |  |  |  |  |  |  |  |
| Carlsson^10^, 1995, Sweden | Anaesthetist & Nurses | Propofol | EGD | 45 | 4 | 0 | 0 | 0 | 18±8 |  | 35 | 37 |  | 3 |
|  |  | Midazolam |  | 45 | 4 | 0 | 0 | 0 | 30±23 |  | 32 | 33 |  |  |
| Chin^11^, 1992, Singapore | Unknown | Propofol | EGD | 31 | 16 | 0 | 0 |  |  |  | 18 | 30 |  | 2 |
|  |  | Midazolam |  | 29 | 17 | 0 | 0 |  |  |  | 28 | 28 |  |  |
| Patterson^15^, 1991, Ireland | Physician | Propofol | EGD | 21 | 4 | 0 | 0 | 0 |  |  | 3 |  |  | 2 |
|  |  | Midazolam |  | 19 | 5 | 0 | 0 | 0 |  |  | 13 |  |  |  |

Abbreviations: N, number of patients; HPX, hypoxia (SaO2 < 90%); HPT, hypotension (SBP < 90 mmHg); AR, arrhythmia; AP, apnea; RT, recovery time (min); PARS, postanesthesia recovery score; AM, amnesia; SL, sedation level; PC, patient cooperation (evaluated by visual analogue scales).
